# The Hidden Costs of a Free Caesarean Section Policy in West Africa (Kayes Region, Mali)

**DOI:** 10.1007/s10995-015-1687-0

**Published:** 2015-02-10

**Authors:** Marion Ravit, Aline Philibert, Caroline Tourigny, Mamadou Traore, Aliou Coulibaly, Alexandre Dumont, Pierre Fournier

**Affiliations:** 1 Global Health Axis, University of Montreal Hospital Research Center (CRCHUM), Montreal, Canada; 2 Department of Biology, University of Ottawa, Ottawa, Canada; 3 Interdisciplinary Research Centre on Well-being, Health, Society and Environment (Cinbiose), University of Quebec in Montreal, Montreal, Canada; 4 URFOSAME, Referral Health Center of the Commune V, Bamako, Mali; 5 Research Institute for Development, Paris, France

**Keywords:** EmONC, Caesarean section, Maternal health, Fee exemption, Expenses of care, West Africa

## Abstract

The fee exemption policy for EmONC in Mali aims to lower the financial barrier to care. The objective of the study was to evaluate the direct and indirect expenses associated with caesarean interventions performed in EmONC and the factors associated with these expenses. Data sampling followed the case control approach used in the large project (deceased and near-miss women). Our sample consisted of a total of 190 women who underwent caesarean interventions. Data were collected from the health workers and with a social approach by administering questionnaires to the persons who accompanied the woman. Household socioeconomic status was assessed using a wealth index constructed with a principal component analysis. The factors significantly associated with expenses were determined using multivariate linear regression analyses. Women in the Kayes region spent on average 77,017 FCFA (163 USD) for a caesarean episode in EmONC, of which 70 % was for treatment. Despite the caesarean fee exemption, 91 % of the women still paid for their treatment. The largest treatment-related direct expenses were for prescriptions, transfusion, antibiotics, and antihypertensive medication. Near-misses, women who presented a hemorrhage or an infection, and/or women living in rural areas spent significantly more than the others. Although abolishing fees of EmONC in Mali plays an important role in reducing maternal death by increasing access to caesarean sections, this paper shows that the fee policy did not benefit to all women. There are still barriers to EmONC access for women of the lowest socio-economic group. These included direct expenses for drugs prescription, treatment and indirect expenses for transport and food.

## Background

A recent report showed that the total number of maternal deaths decreased by 45 % from 523,000 in 1990 to 289,000 in 2013. Nevertheless, more than 60 % of these deaths occurred in sub-Saharan Africa, and it has been estimated that only 19 countries had achieve Millennium Development Goal 5 by 2013 [1].

One of the essential elements for reducing maternal mortality is to increase access to emergency obstetric and neonatal care (EmONC) available in health care centres (basic EmONC) and in referral hospitals (comprehensive EmONC) [2]. The issue of access to EmONC for women is major, given that around 15 % of pregnant women experience complications [3, 4] and that 3.6–6.5 % of women require caesareans in childbirth in West Africa [5]. Indeed, in Africa most caesarean sections are performed in an EmONC [5]. Some studies have shown that many women delay seeking care or decide against going to a health facility out of fear of incurring considerable expenses [6–8]. Improving rapid access to caesarean increases its efficacy and women’s chances of survival [9]. It has been shown that access to caesarean sections is directly influenced by household wealth [10]. The cost of a caesarean episode to the user can be very high, up to ten times higher than for a normal delivery, and caesareans are even considered being the most expensive emergency obstetric intervention [8, 11–13]. However, some financial measures help improve women’s access to emergency obstetric care, such as insurance plans, conditional transfers, voucher systems, equity funds, cost sharing, or even total abolition of user fees [10, 14].

Some countries, such as Burkina Faso, Tanzania, Morocco, Ghana, and Senegal, have recently implemented user fees exemption policies for maternal health care services [15–19]. User fees exemption policies in EmONC for caesarean interventions consist in total fee exemption of the intervention, post-intervention if needed, laboratory tests as well as a kit including medications and basic surgical equipment. However, these policies have not been always effective. In Tanzania, three-quarters of women have to pay for deliveries despite the existence of user fees exemptions. In Morocco where caesareans are free, households still have to pay between 169 USD in a University Hospital and 291 USD in a governmental service managed autonomously hospital in 2010 [17]. In Bangladesh, even though maternity services in hospitals are supposed to be free, households must pay for deliveries (31.9 USD) and caesareans cost nearly four times higher (117.5 USD) [20]. Finally, the average cost of a caesarean in Pakistan is 162 USD while maternal health care is supposedly free [18].

While Mali’s maternal mortality ratio (MMR) has significantly decreased over the past 30 years, it remains high [21]. In 2008, the reported MMR was 670 maternal deaths per 100,000 live births, whereas for the rest of West sub-Saharan Africa the MMR was 629 [21]. In the period 1996–2003, the rate of caesarean coverage in Mali was only 1.2 % at the national level and its distribution was uneven with 3.5 % in urban areas and 0.5 % in rural areas [22].

To reduce maternal mortality Mali implemented two policies for improving access to EmONC. First, in 2002, the government launched a referral evacuation system (RES) to improve access to EmONC. This system included improvements in the quality of EmONC, transportation, communication systems, and a community funding mechanism [23]. It has been shown to be effective in increasing EmONC coverage and in reducing mortality among the women treated [24]. Second, in 2005, Mali introduced user fees exemptions for caesareans in EmONC. This measure covered the surgical procedure and pre-operative examinations, the surgical kit and postoperative treatment (standardized set of [23] products and medications), and hospitalization [25]. A 2011 USAID report showed that after the introduction of the user fees exemption in Mali, the rate of caesarean coverage increased while the rates of post-caesarean maternal and neonatal mortality decreased, even tough no causal relationship was demonstrated [23]. The continued high cost of transportation has been a significant barrier to access to care and the RES needed to be reinforced [23]. Some shortcomings have been showed in the policy’s conception and implementation, particularly with regard to the standardized caesarean kit, the availability of blood for transfusions, and in the policy’s sustainability.

As the present study is part of a larger project conducted on the impact of the three types of delay on institutional maternal mortality and the expenses related EmONC in the Kayes region in Mali (Causes et effets du premier retard sur la létalité des urgencies obstétricales dans la region de Kayes (Mali) 2008–2011), we decided to assess any expenses that were associated with a caesarean episode in the context of user fees exemption.

## Methods

### Study Population and Sample

The study population in the larger project consisted of 484 women, who were both maternal deaths and near-misses and experienced a caesarean section in the latter case–control study. A near-miss was defined as a pregnant women who survived to severe medical complications such as prenatal or postpartum hemorrhage, severe pre-eclampsia, eclampsia, miscarriage, or uterine rupture of the uterus or obstructed. Cases were restricted to the following four obstetric complications—hemorrhage, eclampsia, postpartum infection and uterine rupture—as their first signs were easily recognized by the community and health professionals when following the national clinical guideline. When a woman had experienced two or more complications, the most serious or the lethal one was selected. Since the criterion of inclusion in the present study included only women who delivered by caesarean in EmONC, a total of 190 women were finally selected (95 deceased and 95 near-misses). For consistency purposes, one same sampling design was conducted for near-misses and deceased women. The interviewer traced back the woman’s steps and conducted the investigation starting in the last place the woman was treated (district health center or regional hospital), moving on to the community health center (CSCom) and ending at the woman’s household. Medical records were recorded from the health professionals who treated the woman. A social interview was conducted with the head of the household and/or any household member who was present during the caesarean episode. The questionnaire administered during the interview recorded socio-demographic data, information on the episode, such as transport time, and any data of expenses related to caesarean intervention.

### Wealth Index

Because household incomes were not available, the socio-economic status was estimated using a wealth index based on assets. This estimated wealth index was obtained using principal components analysis (PCA), as commonly performed in some other studies [26–28]. This index has been calculated based on the possession (Yes/No) of eight variables assets as follow: the household’s items (cellular phone, stereo, motorcycle), the quality of the home’s building materials (roof and floor), ownership of the house, cattle for commerce and cattle for consumption. The first two dimensions of the PCA explained 55.26 % of the total variation after varimax rotation (37.14 % for dimension 1 and 18.12 % for dimension 2). Only the dimensions with an eigenvalue greater than 1 were retained for further analyses. For each selected dimension, responses to the items were weighted and averaged to create an overall score and thus an integrative wealth index was calculated. The wealth index was used as a categorical variable for analysis purposes (three categories).

### Data Analysis

Although the women were paired in the larger project (Causes et effets du premier retard sur la létalité des urgencies obstétricales dans la region de Kayes (Mali)), the present analyses based on the caesarean sections used the variable near-misses versus deceased women as a covariate in statistical analyses. One woman was excluded from the analyses as an outlier due to extremely high treatment expenses (356,000 FCFA-754 USD) (1 USD = 472 FCFA, 2008–2011 average).

Four categories of expenses were considered in the statistical analyses, as follow: total amount of expenses, expenses for treatment, transportation and other expenses. For a total of six women for who only the total amount of expenses was available, a mean imputation procedure was computed to replace missing values in the other categories of expenses such as treatment, transportation and others.

**Fig. 1:**
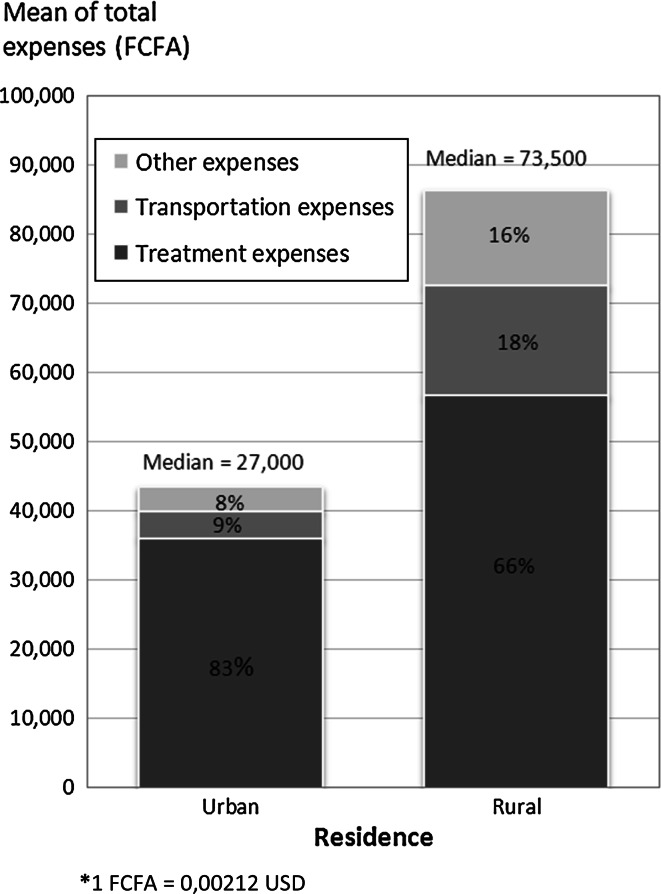
Mean proportion of expenses by residence (n = 190, FCFA*)

**Fig. 2:**
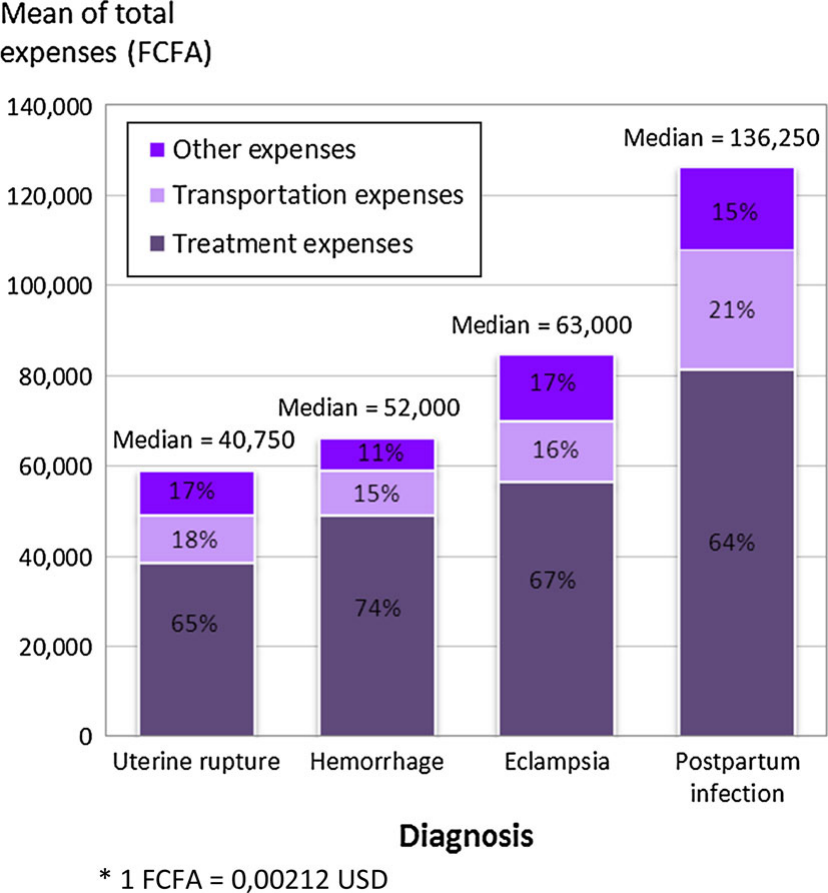
Mean proportion of expenses by diagnosis (n = 190, FCFA*)

**Fig. 3:**
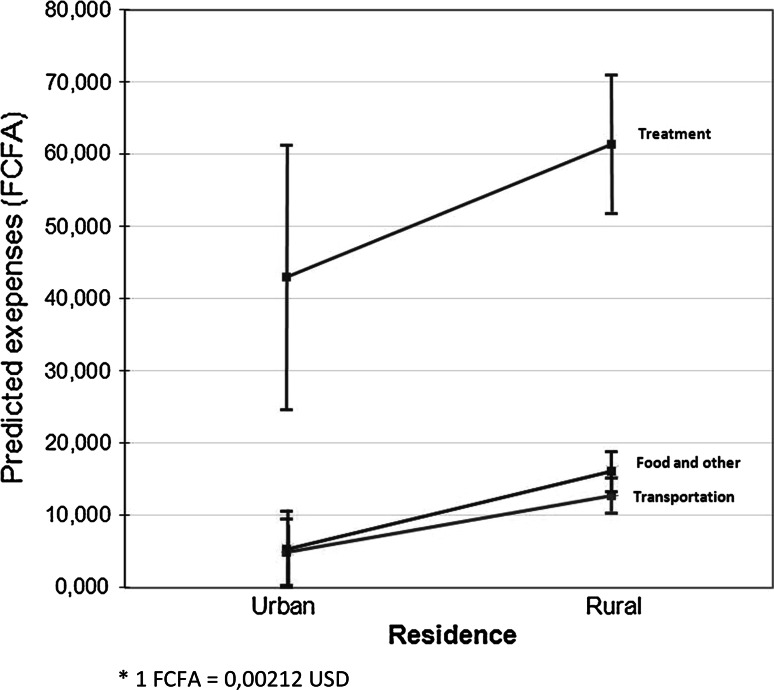
Marginal predicted values of expenses by residence with 95 % confidence interval (n = 190, FCFA*)

**Fig. 4:**
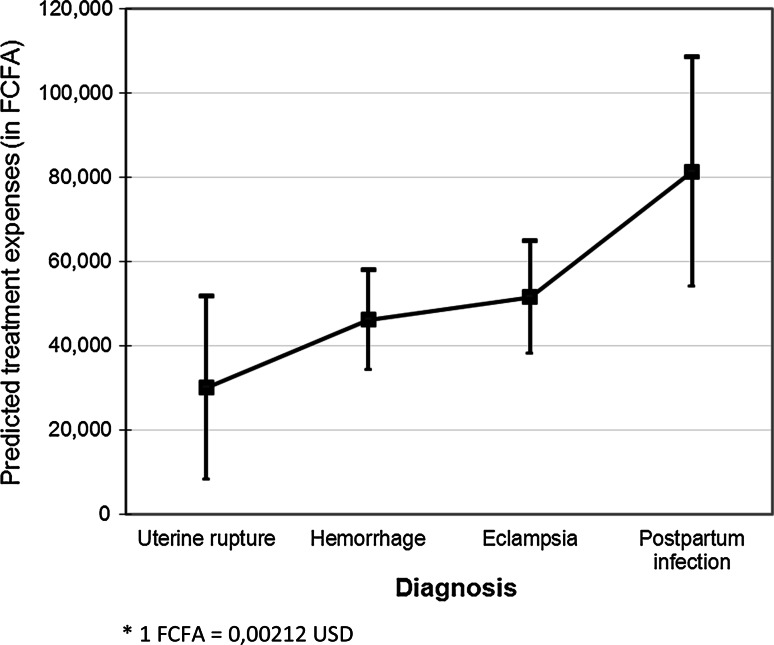
Marginal predicted values of treatment expenses with 95 % confidence interval (n = 190, FCFA*)

Descriptive analyses were used to display the profile of factors Figs. 1, 2 and Tables 1, 3, 4. For the Figs. 3 and 4, marginal predicted values, that are the expected value of a typical observation from some level of a categorical factor when all the other factors have been set to neutral values, were used. Mann–Whitney *U* test was used to compare the four categories of expenses between women according to their residence or the diagnosis. Following a forward stepwise procedure, a series of multivariate linear regression analyses were completed to identify which factors were significantly associated with each category of expense. Outcomes of the caesarean episode (near-misses versus deceased women), diagnosis, permanent residence of the parturient, and wealth index were considered as potential factors and In all regression analyses, the absence of collinearity among variables was assessed with the tolerance and variance inflation factor (VIF) measures. Homoscedasticity of residuals was also verified. Finally, any influential data was tested with Cook’s distance.

**Table 1:** Characteristics of the two-dimension of the principal component analysis (factorial loading of selected items, eigenvalues, % of variance explained and Cronbach’s alpha values on each dimension are provided), varimax rotation

List of significants items	Factorial loading of items selected on each dimension	
	Dimension 1	Dimension 2
	Eigenvalue	Eigenvalue
	3.381	1.610
	% of explained variance (37.57 %)	% of explained variance (17.89 %)
	Cronbach’s	Cronbach’s
	α = 0.78	α = 0.75
Owning a motorcycle in the household	0.663	
Owning a TV in the household	0.815	
Owning a stereo in the household	0.775	
Owning a mobile phone in the household	0.681	
Manufacturing equipment from the floor of the house	0.783	
Manufacturing equipment from the roof of the house	0.729	
Owning cattle for commerce in the household		0.896
Owning cattle for consumption in the household		0.875

The limit of significance for statistical tests was set at ≤0.05.

Analyses were performed using SPSS version 20 (SPSS Inc., Chicago, IL) and JMP software package version 5.1 (SAS institute INC, Cary, NC, USA).

### Ethical Approval

This study was approved by the research ethics committee of the University of Montreal Hospital Research Centre (CRCHUM, Canada) and by the ethics committee of the Faculty of Medicine, Pharmacy and Dentistry of the University of Bamako (Mali).

## Results

### Wealth Index and Sample

Table 1 shows the characteristics of the two-dimension of the principal component analysis.

In decreasing order the outcome episode was hemorrhage (41.4 %), eclampsia (36.1 %), uterine rupture (13.6 %), or postpartum infection (8.9 %). For each category the same proportion was found in deceased women or near-misses (data not shown). On average, the total time of transportation from home to the site of the caesarean intervention exceeded 2 h (median = 1 h, 19 min). Finally, more than three quarters (77.5 %) were residing in urban areas.

### Analysis of Expenses

Women in the Kayes region spent on average 77,017 FCFA (163 USD) (median = 61,250 FCFA-130 USD) for the caesarean episode, of which 70 % was for treatment, 14 % for transportation, and 16 % for food and other items (Table 2). Even though the caesarean was free at the point of service, 91 % of the women had to pay for the treatment with a cost reaching in average 59,241 FCFA (126 USD). In general, a caesarean for a near-miss did cost twice as much than that for a deceased women.

**Table 2:** Characteristics of women who had caesareans (n = 190) and of their expenses by category in FCFA*, Kayes, Mali (2008–2011)

Characteristics	N (%)	Total expenses	Treatment expenses	Transportation expenses	Other expenses
		Mean ± standards deviations (median)	Mean ± standards deviations (mean proportion of total expenses)		
*Deceased/Near-misses*					
Deceased	95 (50 %)	51,197 ± 48,770 (32,000)	35,850 ± 38,288 (72 %)	12,142 ± 16,354 (23 %)	3,205 ± 7,512 (5 %)
Near-misses	95 (50 %)	102,838 ± 76,146 (90,000)	72,031 ± 64,954 (67 %)	9,554 ± 12,508 (10 %)	21,253 ± 20,916 (23 %)
*Diagnosis*					
Eclampsia	69 (36.3 %)	84,705 ± 78,333 (63,000)	56,415 ± 62,111 (67 %)	12,149 ± 15,817 (16 %)	16,141 ± 22,777 (17 %)
Hemorrhage	79 (41.6 %)	66,198 ± 56,802 (52,000)	49,946 ± 48,660 (74 %)	8,384 ± 12,828 (15 %)	7,868 ± 12,103 (11 %)
Postpartum infection	16 (8.4 %)	126,350 ± 55,392 (136,250)	85,688 ± 48,771 (64 %)	20,663 ± 14,311 (21 %)	20,000 ± 18,000 (15 %)
Uterine rupture	26 (13.7 %)	59,131 ± 69,216 (40,750)	39,969 ± 60,329 (65 %)	8,846 ± 14,150 (18 %)	10,315 ± 16,392 (17 %)
*Residence*					
Urban	42 (22 %)	43,569 ± 47,002 (27,000)	38,390 ± 43,759 (83 %)	1,119 ± 1,052 (9 %)	4,060 ± 6,879 (8 %)
Rural	148 (78 %)	86,510 ± 71,161 (73,500)	58,353 ± 58,613 (66 %)	13,609 ± 15,434 (18 %)	14,548 ± 19,581 (16 %)
*Wealth index*					
Poorest tercile	67 (35.3 %)	69,715 ± 50,918 (5,000)	45,360 ± 38,646 (64 %)	15,735 ± 16,713 (24 %)	8,619 ± 12,680 (12 %)
Intermediate tercile	62 (32.6 %)	92,622 ± 92,539 (65,458)	67,572 ± 76,081 (74 %)	10,206 ± 13,822 (12 %)	14,844 ± 21,720 (14 %)
Wealthiest tercile	61 (32.1 %)	69,178 ± 54,679 (63,000)	49,508 ± 46,054 (71 %)	6,133 ± 10,874 (11 %)	13,537 ± 18,757 (18 %)
*Total transport time*					
Less than 1 h	70 (36.8 %)	64,816 ± 66,413 (45,500)	50,051 ± 54,060 (77 %)	3,600 ± 6175 (9 %)	11,164 ± 19,759 (14 %)
Between 1 and 2 h	48 (25.3 %)	70,917 ± 58,837 (56,000)	49,020 ± 46,614 (68 %)	9,793 ± 11,297 (16 %)	12,104 ± 16,048 (16 %)
More than 2 h	72 (37.9 %)	92,947 ± 74,849 (79,000)	61,001 ± 63,577 (63 %)	18,599 ± 18,186 (23 %)	13,348 ± 17,875 (14 %)
Total		77,017 ± 68,826 (61,250)	53,940 ± 56,182 (70 %)	10,848 ± 14,578 (14 %)	12,229 ± 18,097 (16 %)
% of women with no expenses		4 % (n = 8)	9 % (n = 17)	34 % (n = 64)	51 % (n = 97)

* 1 FCFA = 0,00212 USD

For the caesarean episode the women living in rural areas spent significantly more money for treatment, transportation, and food than did the urban women (Fig. 1).

Figure 2 presents the different categories of expenses according to the diagnosis. On average, the total costs incurred for treatment and transportation were significantly higher for cases of postpartum infection than for other complications. Expenses for food and other items were on average higher for cases of eclampsia compared with cases of hemorrhage, and for cases of postpartum infection compared with cases of hemorrhage and of uterine rupture, to a significantly extent (*p* < 0.05).

### Factors Associated with Expenses

Being a near-miss, presenting a postpartum infection, and/or residing in a rural area were factors associated with higher expenses. The poorest households spent less than did the wealthiest ones on food and in total expenses. Longer travel times resulted in higher transportation costs were associated with higher overall costs (Table 3).

**Table 3:** Factors associated with the expenses of women who had caesareans in Kayes region

	Total expenses			Treatment expenses			Transportation expenses			Other expenses		
	Beta ± standard error	Std beta^a^	*p* value^b^	Beta ± standard error	Std beta	*p* value	Beta ± standard error	Std beta	*p* value	Beta ± standard error	Std beta	*p* value
Outcome of the episode												
*Reference: deceased*												
Near-misses	27,788 ± 4,292	0.405	0.000**	19,028 ± 3,774	0.339	0.000**	–	–	–	8,763 ± 1,079	0.485	0.000**
Diagnosis												
*Reference: uterine rupture*												
Eclampsia	3,790 ± 7,171	0.037	0.598	−712 ± 6,438	−0.008	0.912	1,187 ± 1,578	0.055	0.453	2,523 ± 1,810	0.094	0.165
Hemorrhage	−12,245 ± 7,303	−0.123	0.095	−6,118 ± 6,450	−0.075	0.344	−425 ± 1,601	−0.020	0.791	−5,590 ± 1,829	−0.213	0.003**
Postpartum infection	43,905 ± 11,517	0.299	0.000**	29,029 ± 10,396	0.242	0.006**	5,567 ± 2,536	0.179	0.029*	7,778 ± 2,910	0.201	0.008**
Residence												
*Reference: urban*												
Rural	14,720 ± 5,740	0.178	0.011*	9,227 ± 4,721	0.137	0.052	3,914 ± 1,225	0.223	0.002**	5,413 ± 1,398	0.249	0.000**
Wealth index (terciles)												
*Reference: wealthiest tercile*												
Poorest tercile	−15027 ± 6677	−0.179	0.026*	–	–	–	–	–	–	−3,929 ± 1,621	−0.179	0.016*
Intermediate tercile	17,652 ± 6,013	0.207	0.004**	–	–	–	–	–	–	2,346 ± 1,407	0.104	0.121
Total travel time	7,766 ± 2,417	0.227	0.002**	–	–	–	2,565 ± 508	0.354	0.000**	–	–	–
R^2^	0.34			0.17			0.26			0.39		
N	190			190			190			190		

^a^Standardized beta, ^b^ Student’s *t* test, * *p* < 0.05, ** *p * < 0.01

Figure 3 shows that, over and above the influence of other factors, all types of predicted expenses were higher for residents of rural areas.

### Expenses for Treatment

Independently of the characteristics of women and their households, the expenses for treatment represented the largest portion of expenses with an average of 70 % of total expenses. However, the treatment expense increased with the health facility level. Indeed, the cost of a caesarean undergone at the regional hospital was greater than that in a district hospital (data not shown).

Figure 4 shows the marginal predicted values of treatment expenses for each type of complication when controlled for the following covariables: outcome of the episode (near-miss or death), residence (rural or urban), wealth index (poorest, intermediate or wealthiest tercile) and total transport time (hours)). The marginal predicted values of treatment expenses varied with complication, 29,988 FCFA (64 USD) for an episode of uterine rupture, 46,070 FCFA (98 USD) for a hemorrhage, 51,476 FCFA (109 USD) for eclampsia, and 81,217 FCFA (172 USD) in the case of a postpartum infection.

Treatment expenses were subdivided into subcategories of medications and therapeutic indications. A total of 13 subcategories were found: transfusions, antibiotics, antihypertensives, anticonvulsants, antispasmodics, antimalarials, analgesics, uterine myocontractants, laparotomy, technical procedures, diagnostic testing (blood tests, urinalysis, ultrasound), supplementation, and intravenous fluid therapy. Table 4 clearly shows that transfusions, antibiotics, and antihypertensives increased the total amount of treatment expenses. Receiving transfusions or being administered one or more antibiotics had a major effect on the women’s expenses. Depending on the amount of blood transfused, these women’s expenses were 10–20 times higher than for women who did not receive transfusions. Women who received one antibiotic had significantly higher expenses than those who received none, and those who received two or more antibiotics had higher expenses than did women who received only one. Similarly, women who received two antihypertensive medications or more presented higher expenses on average than did women who received none (Table 4).

**Table 4:** Treatment expenses for women by type of treatment in FCFA*, for women with positive expenses (n = 174)

	Total treatment costs (in FCFA)	
	N	Mean ± standards deviations
Transfusion		
None	117	58,142 ± 56,629
1–2 bags	46	55,211 ± 48,849
3–6 bags	11	114,751 ± 107,777
Antibiotics		
None	56	38,538 ± 45,766
One	39	49,664 ± 36,672
Two or more	79	82,400 ± 71,077
Antihypertensives		
None	117	56,416 ± 57,513
One	37	58,722 ± 47,643
Two or more	20	91,562 ± 86,674
Other treatment (none of the 3 categories above)	21	28,500 ± 24,276
One of the 3 categories above	77	51,654 ± 51,118
Two or more of the 3 categories above	76	79,087 ± 69,761

* 1 FCFA = 0,00212 USD

## Discussion

The vast majority of the women (91 %, or 174 out of 190) did not beneficiate from the existing fee exemption policy and paid for their caesarean intervention for EmONC. In some cases, these expenses were very high; for eight women they were above 200,000 FCFA (424 USD) and they could even exceed 350,000 FCFA (742 USD). A study carried out in the same region of Kayes demonstrated that the fee exemption policy did not prevent from catastrophic expenses women who had a caesarean and women who did not during an obstetric complication [29].

Women’s expenses varied depending on of the episode (near-miss or death), residence (rural or urban) and total transport time (hours), wealth index (poorest, intermediate or wealthiest tercile) the site of the caesarean episode, the type of complication and treatment. Indeed, the family of the deceased women spent less on average than did the ones of the near-misses. However our data prevent us to determine whether a limited financial capacity impeded women’s survival or whether expenses were lower due to a shorter treatment period as they deceased.

Transportation costs remained high despite the existence of the RES, and they impeded access to EmONC. The RES was intended to lower transportation expenses and reduce inequalities. Unfortunately, this system was not fully functional, partly because the collection on the solidarity funds, which ensured the system’s operations, was only 21 % in 2009, with considerable heterogeneity among the regions [30]. Transportation costs was higher for women living in rural areas and their impacts were greater on the poorest women. Women in the poorest tertile were women who spent the least on their caesarean episode, due maybe to the low spending on food or low costs generated by the intervention according to the type of complication. It remains possible that the low costs for treatment might indicate that they did not receive all the necessary services.

Our findings are in concordance with results found in another study conducted in Mali [23]. They showed that despite the caesarean fees exemption policy, inequalities in access to EmONC persisted among the different economic groups. In particular, the costs of transport were higher for the poorest households, who most often lived in rural areas [31].

The site of treatment also influenced the level of expenses; women whose caesareans were performed at the regional hospital paid much more than did women whose intervention occurred in a district hospital. We suggest the better quality of services and well reputation in regional hospital might have been responsible for higher treatment expenses.

The women who had a postpartum infection after the caesarean intervention paid more than did women with other complications, especially for treatment. We could be explained by the fact that postpartum infections often require long hospitalization and medications, especially antibiotics, and for some women even a second intervention. Such complication might have negative impacts on the poorest households (62.5 % of postpartum infections) and women living in rural areas, who are often considered as the poorest (100 % of postpartum infections).

Treatment expenses were by far the most substantial, and there might be many reasons for these high costs. The provisions of the caesarean fees exemption were not clearly defined, and it was difficult to specify at what point it took effect. The fee exemption covered only the intervention itself and any immediate post-intervention complications. On the other hand, once the woman returned home, any other complication that might have arised at the household’s expense. Thus, households might incur considerable expenses both before and after a caesarean intervention episode is better [30].Women living in rural areas paid more for treatment than other, probably because they were more vulnerable and less educate Finally and although the fees exemption for EmONC comes with standardized kits for caesareans, it happens that only one type of antibiotic (amoxicillin) and very little pain medication were provided. When medication is needed it was an extra fee for the household [30] (see Box 1).

**Box 1:** Example of the consequences of the extra fees paid by households for medication

The woman arrived at the health centre around 1:00 p.m. and was evacuated at around 8 p.m. to the district hospital for prolonged dystocic labour. After an unsuccessful attempt at forceps delivery, she underwent a caesarean at 11:00 p.m. After surgery, she developed an infection, not responsive for many days to several antibiotics. She also received three blood transfusions for anaemia. While the household did not have to pay for food and transportation, treatment at the health centre cost 6,000 FCFA (13 USD) and at the district hospital, 350,000 FCFA (742 USD)	

Another cause of major expenses relates to blood transfusions. In Mali, blood tests and transfusion supplies are free only in the capital of Bamako. Blood is likely missing in the bank of the hospital as shown in other studies [32]. In that case, if no donor is available among the people accompanying the patient or the family, a paying service from an informal network is generally used. The price per bag of blood can range from 30,000 to 50,000 FCFA (64–106 USD). Box 2 presents an example of the tragic consequences of problems related to the availability of blood.

**Box 2:** Example of the consequences of problems related to the availability of blood for households

At the end of her pregnancy, the woman noticed bleeding and decided to go to the hospital the same day. She arrived at 11:20 a.m. The physician diagnosed a retroplacental hematoma and performed at 11:40 a.m. The woman delivered a fresh stillborn child. During the intervention, she began hemorrhaging profusely. The physician had requested three bags of blood, but was unsuccessful in getting these. At around 7:00 p.m, two bags of blood were found. Finally, the woman died the following morning around 9:00 a.m. The hospital treatment cost 65,000 FCFA (138 USD) and the food was provided by the family. To cover the costs, the husband had to sell a refrigerator, which he had been using to sell ice and cold drinks in his village.	

The strength of our paper is that very few studies have estimated the expenses and the factors associated with these expenses for a caesarean intervention in a context of user fees exemption [17, 18]. Our findings are similar to results recently reported in Morocco and Pakistan [17, 18]. Women who had undergone caesareans in the regional or provincial hospital of the Fez district had spent, on average, 169 USD.

However, our study shows some limitations. There maybe a recall bias in sampling treatment expenses data. During the interview a questionnaire was administered to the family members who were present during the caesarean episode that may had occurred months ago. Although the four complications were assumed to be easily recognized by health professionals with national clinical guidelines, it did not preclude from diagnostic error. Second, the household wealth index remains an approximation of the socio-economic status and relates only to our sample. Third, our sample size was limited to 95 persons in each group. Even though this number is reasonable for regression analyses, it may limit the power analysis. Fourth, women who died following a caesarean were over-represented in the sample; they made up 50 % of the sample, whereas if compared to a study in Mali and Senegal, the mortality rate of women following a caesarean was 1.7 % [33]. Since the deceased women incurred fewer costs than did the near-misses, expenses overall maybe under-estimated. Finally, our sample may be not well representative of the national population but they are at the regional level. Indeed, our sample consisted of women with various sociodemographic characteristics, coming from a variety of geographic areas and receiving caesarean interventions at several levels of care (district or regional hospital). However, we believe that our findings could be generalized to regions outside the capital of Mali or comparable low-income countries, while keeping in mind that the expenses were probably under-estimated.

## Conclusions

Although abolishing fees for delivery and caesarean interventions in Mali plays an key role in reducing maternal and newborn death by increasing access to EmONC, the present study shows that fee exemption may not benefit to all women. Even though the EmONC fee exemption policy has been in place since 2005, a total of 91 % of women still have to pay for treatment.

Among the direct expenses were included costs for drug prescription, and treatment (including transfusion). Transportation and food were shown as the most important indirect costs. These indirect costs represent an unpredictable expenses for a household to cope with. The caesarean fees exemption policy and the RES are solutions to improve women’s access to emergency obstetric care, but they are not enough. Although the fee exemption policy aims to improve maternal health care accessibility and reduce inequalities between the poorest and the wealthiest households, our findings clearly show there are still important economical access barriers for the poorest ones. An important step for Mali towards realizing the Millennium Development Goal No. 5 will be to cover all expenses.
